# Influence of Homogenization on Microstructural Response and Mechanical Property of Al-Cu-Mn Alloy

**DOI:** 10.3390/ma11060914

**Published:** 2018-05-29

**Authors:** Jian Wang, Yalin Lu, Dongshuai Zhou, Lingyan Sun, Renxing Li, Wenting Xu

**Affiliations:** 1 School of Materials Engineering, Jiangsu University of Technology, Changzhou 213001, China; wjhjj1217@163.com (J.W.); zhouds@jsut.edu.cn (D.Z.); linnsun@163.com (L.S.); jxlrx@jsut.edu.cn (R.L.); 2014560009@jsut.edu.cn (W.X.); 2 Key Construction Laboratory of Green Forming and Equipment from Jiangsu Province, Changzhou 213001, China

**Keywords:** Al-Cu-Mn alloy, homogenization, microstructures response, mechanical property

## Abstract

The evolution of the microstructures and properties of large direct chill (DC)-cast Al-Cu-Mn alloy ingots during homogenization was investigated. The results revealed that the Al-Cu-Mn alloy ingots had severe microsegregation and the main secondary phase was Al_2_Cu, with minimal Al_7_Cu_2_Fe phase. Numerous primary eutectic phases existed in the grain boundary and the main elements were segregated at the interfaces along the interdendritic region. The grain boundaries became discontinuous, residual phases were effectively dissolved into the matrix, and the segregation degree of all elements was reduced dramatically during homogenization. In addition, the homogenized alloys exhibited improved microstructures with finer grain size, higher number density of dislocation networks, higher density of uniformly distributed θ′ or θ phase (Al_2_Cu), and higher volume fraction of high-angle grain boundaries compared to the nonhomogenized samples. After the optimal homogenization scheme treated at 535 °C for 10 h, the tensile strength and elongation% were about 24 MPa, 20.5 MPa, and 1.3% higher than those of the specimen without homogenization treatment.

## 1. Introduction

Al-Cu alloy has extensive applications in the aerospace industry and in varied structural transportation components for its excellent characteristics of elevated specific strength, good high-temperature performance, favorable weldability, and outstanding corrosion resistance [1,2,3,4]. Thus, the microstructures and properties of this alloy have been investigated in many studies due to its significance in aviation and aerospace fields [5,6,7,8]. Mazutina et al. [6] studied the effect of deformation temperature (from 250 to 475 °C) on the microstructure evolution of the coarse-grained aluminum alloy 2219 during equal channel angular pressing. Prabhu et al. [7] demonstrated the effect of ageing time on the conductivity and mechanical properties of Al-Cu alloy round bars with various diameters. The studies showed that the precipitates coarsened with increasing ageing time, but precipitate segregation was not found in the microstructure for any cases of round bar diameter and ageing time combinations.

The semi-solid casting [9], deformation process [10], heat treatment [11], and many other methods [12] can all determine the phase transformation and microstructure evolution of aluminum alloys. However, uneven cooling rate in different regions of the ingots during direct chill (DC) casting results in the formation of severe interdendritic segregation and coarse intermetallics, which gravely affect the followed processabilities and hampers service performance [13,14,15]. Furthermore, features of the microstructure are extremely difficult to alter during the subsequent plastic processing and heat treatment. So, it is necessary to carry out homogenization treatment directly for as-cast aluminum alloys before the plastic processing, in order to reduce or eliminate enough of the eutectic intermetallic particles to improve the plastic deformation of the alloys and hence product performance. The emerge of eutectic intermetallic particles has a negligible impact on processing properties due to the microsegregation of Cu, Fe, and Mg elements and other alloying elements or intermetallics [16]. The as-cast microstructures and the evolution behaviors of the eutectic phases during homogenization in Al-Cu alloys have been researched in recent years [17,18,19,20]. Du et al. [17] investigated the impact of homogenization on the microstructures and mechanical properties of 2A50 aluminum alloy prepared by liquid forging. Liu et al. [18] revealed that the residual phases in alloy ingot are dissolved into the matrix gradually, grain boundaries become sparse, and the distribution of all the elements becomes more homogenized by applying the homogenization treatment, which can be described by a constitutive equation in an exponential function. However, the study of the as-cast microstructure of 2219 aluminum alloy and its evolution behaviors during homogenization have not been reported in previous investigations. Furthermore, there is no comprehensive research on the homogenized microstructures of Al-Cu-Mn (2219) alloys. The objectives of the present work were to ascertain the details of the effect of homogenization treatment on the microstructural response and the capability of the homogenization processes to improve the mechanical properties. So then, it can be applied in relative experiments as well as in actual industrial production of the alloys.

## 2. Experimental Procedures

The 2219 aluminum alloy cast ingots with dimensions Ф 630 mm × 5000 mm used in the present work were produced by direct chill (DC) casting, and the main chemical composition of the studied alloy was Al-6.23 wt % Cu-0.36 wt % Mn-0.18 wt % Zr-0.14 wt % Fe-0.01 wt % Mg. A thickness of 300 mm was sliced from one end of the ingot by sawing machine, and then it was divided into eight equal parts. The samples were treated at 525 °C, 535 °C, 545 °C, or 555 °C for 10 h, respectively. Then, the samples were homogenized at the optimal temperature (535 °C) for 2 h, 6 h, 10 h, or 16 h. All samples were removed from the furnace and immediately quenched into water in order to keep the microstructures after homogenization treatment.

**Figure 1:**
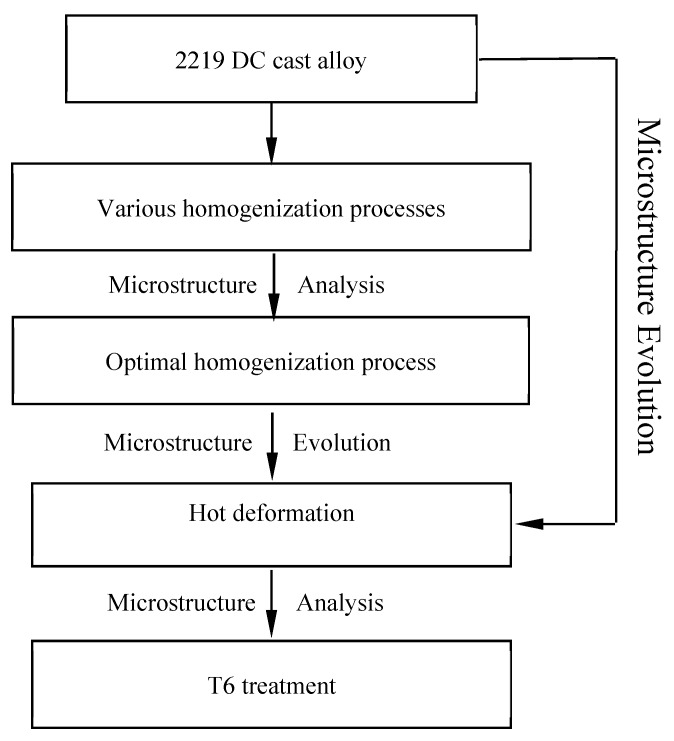
Experimental procedures applied to the AA2219 direct chill (DC) cast alloy.

The DC alloy ingot was first treated by the optimized homogenization process and then multiple axes forged, and finally ring-rolled into a forged ring with size Ф 3375 mm (external diameter)/3200 mm (internal diameter) × 200 mm (height). The complete experimental procedures applied to the AA2219 DC cast alloy are shown in Figure 1. The forged ring was solution treated at 535 °C for 3.5 h, and immediately quenched in water at around 45 °C within 10 s and then aged at 180 °C for 18 h (T6 treatment). The tensile testing was performed on a CMT-5105 electronic universal testing machine operating at a constant crosshead speed of 2.0 mm/min. The tensile properties are the average values of three test bars for each homogenization condition. In addition, the hardness of the as-cast specimen was measured in a HB-3000 Brinell hardness tester, with a load of 30 kg and a 5-mm-diameter ball.

The as-cast, homogenized, deformed, and T6 heat-treated microstructures of the samples were observed by optical microscope (OM) with a ZEISS Axiovert 200 MAT. The chemical composition and dissolution of the intermetallic phases in the as-cast and homogenized samples were characterized using a ZEISS Sigma 500 field emission scanning electron microscope (FESEM, Carl Zeiss, Oberkochen, Germany) equipped with an Oxford Aztec 50 energy dispersive spectrometer (EDS, Oxford Instruments, Abingdon, UK). The values of the results of energy spectroscopic analysis and investigation for each type of phase from at least three different locations are summarized here. To observe the distribution of the elements, line and map scanning analyses were conducted on an EDS measurement. A NETZSCH Proteus differential scanning calorimeter (DSC, Netzsch, Bavaria, Germany) at a constant heating rate of 5 °C/min from 20 °C to 700 °C ascertained the melting temperature of the eutectic phases for different homogenized samples. X-ray diffraction (XRD) analyses were carried out using a Bruker D8 ADVAN/CE X-ray diffractometer (Bruker, Karlsruhe, Germany) to determine the phase constitutions present in as-cast and homogenized materials. To evaluate and quantify the microstructural evolution, all deformed and T6 heat-treated samples of various homogenization conditions were sectioned parallel to the direction of rolling deformation, and then examined by using the electron backscattered diffraction (EBSD) under a ZEISS Sigma 500 field emission scanning electron microscope. The scanning area was 150 × 250 µm^2^ with a scanning step size of 0.1 µm for each deformed sample, and the scanning area was 300 × 380 µm^2^ with a scanning step size of 2 μm for each T6 heat-treated sample. The thin foils were mechanically polished to a thickness of 50 µm and electro-polished with a Tenupol-3 twin-jet by a 20% HClO_4_ and 80% CH_3_OH solution. Then, the double-tilt stage was operated at −25 °C supported by liquid nitrogen and a potential difference of 50–75 V. The substructure evolutions were investigated using a TECNAI G^2^ 20 transmission electron microscope (TEM, FEI Company, Hillsboro, OR, USA) operated at 200 kV.

## 3. Results and Discussion

### 3.1. Characteristics of Large Ingot Prepared by DC Casting

High cooling rate, complex and high content of alloying elements, and non-equilibrium solidification conditions in direct chill (DC) casting inevitably cause severe segregation and the formation of non-equilibrium eutectics and intermetallics [21]. The Cu, Mn, and other alloying elements usually form the uneven distribution of low-melting-point brittle non-equilibrium eutectic compounds or other undissolved intermetallics at the grain boundary, which was proposed by Gao et al. [22]. Coarse intermetallics are extremely difficult to reduce or eliminate by subsequent deformation process and heat treatment, owing to the proximity of composition to the limit of solid solubility in these alloys, which is detrimental to hardenability and fatigue life, and causes variable properties [23,24]. The inevitable features in the as-cast microstructure play a major role in determining the following processing property of aluminum alloys, which can be eliminated by homogenization treatment.

**Figure 2:**
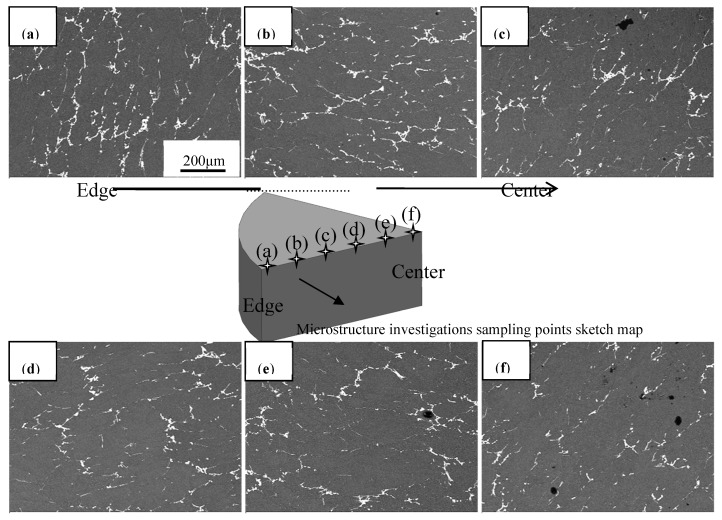
Metallographs of the as-cast 2219 alloy ingot at different distances (mm) along radial direction. (**a**–**f**) metallographic investigations located 10 mm (**a**); 70 mm (**b**); 130 mm (**c**); 190 mm (**d**); 250 mm (**e**) and 310 mm (**f**) from the ingot edge along the radial direction.

Six equidistant discrete samples for metallographic investigations were cut from horizontal cross sections of the ingot located 10–310 mm from the ingot edge along the radial direction, as shown in Figure 2. Coarse microstructure characteristics emerged in the ingot center with a slow cooling velocity. However, smaller grains and some casting defects containing pores and inclusions were located at the ingot edge, dictated by the high cooling rate from the solidification temperature. Obviously, the microstructural inhomogeneity of the larger-size DC 2219 aluminum alloy ingot is due to the different and special cooling conditions at different positions, and is an inevitable phenomenon.

**Figure 3:**
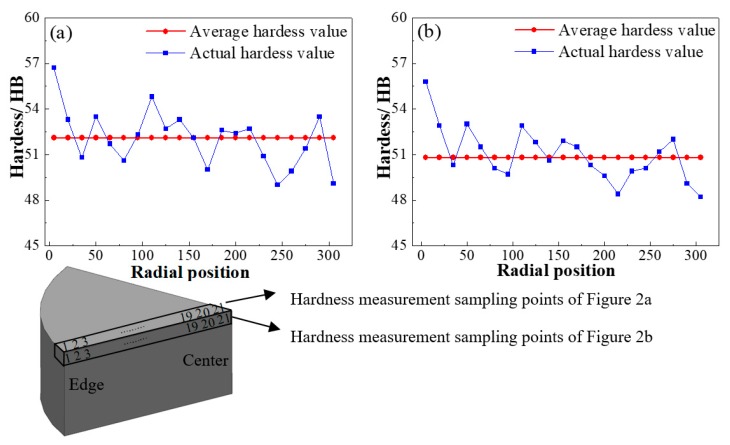
Hardness values in the different sampling points along the radial direction. (**a**) hardness value in the cross-section; (**b**) hardness value in the longitudinal section.

Furthermore, nonuniform microstructures were caused by the inhomogeneous distribution of alloy elements, solidification temperature, and various inclusions, which in turn also create uneven properties. As shown in Figure 3, the hardness measurements were carried out on horizontal sections perpendicular to the solidification direction of the ingot. It can be seen that there was almost no visible trend of the hardness distributions throughout the ingot. Meanwhile, inhomogeneous characteristics of hardness values at different positions could also be found, and the hardness value slowly decreased from the ingot edge to the center. Therefore, a suitable homogenization process is an essential procedure in order to eliminate micro-segregation and obtain as large a supersaturated solubility as possible because of the complexity and inhomogeneity of microstructures and properties during the solidification process.

### 3.2. As-Cast Microstructure

**Figure 4:**
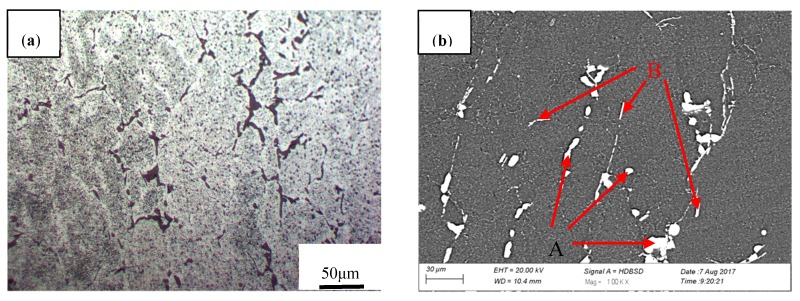

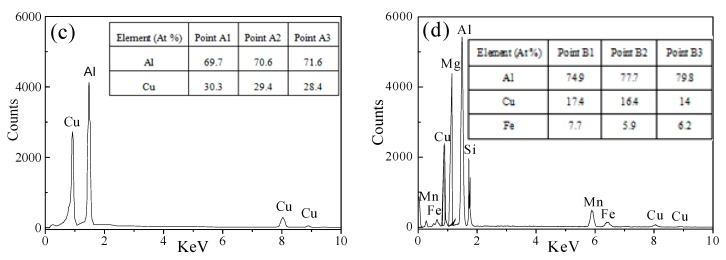
Microstructures and energy dispersive spectroscopy (EDS) results of the as-cast 2219 aluminum alloy: (**a**) Optical microstructure; (**b**) SEM microstructure; (**c**) EDS results for point A; (**d**) EDS results for point B.

The as-cast microstructures of 2219 aluminum alloy are shown in Figure 4. It is a universal phenomenon that as-cast structures consist of primary dendrites of aluminum-rich solid solution (Figure 4a,b). The 2219 alloy ingots had severe microsegregation and the average grain size was about 40–80 μm. There was a large amount of continuous non-equilibrium solidification eutectic elongated along the grain boundaries and at interdendritic regions, which inevitably have negative effects on the strength and toughness of the alloy and produce some negative impacts on the processability and applicability [21]. Different types of eutectic phases are indicated by the arrows in Figure 4b. The EDS results shown in Figure 4c reveal that the gray secondary phases of the block particles (spot A) were determined to be θ phase (Al_2_Cu). The dark gray needle-like phase containing Cu and Fe (spot B and in Figure 4d) was consistent with the stoichiometry of a Al_7_Cu_2_Fe phase. The same characteristics of discrete particles were also shown in other cast structure regions.

**Figure 5:**
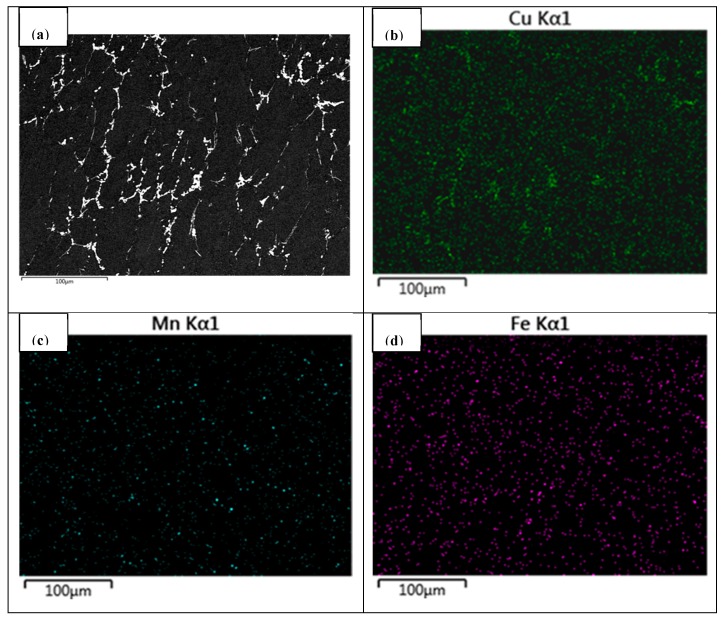
BSEM microstructures and main elements distribution of the as-cast 2219 aluminum alloy: (**a**) BSEM image; (**b**) Cu; (**c**) Mn; (**d**) Fe.

Element segregations could be observed for the main alloying elements, as shown in Figure 5. We can clearly see that main elements Cu, Mn, and Fe were segregated largely in grain boundaries, among which Cu was the most severely segregated element and Mn was the least segregated in grain boundaries. Moreover, Cu was segregated in the form of an Al_2_Cu phase at the grain boundaries, resulting in a much higher Cu content in these regions than that in the matrix. Mn and Fe were presented in almost in the same position in local regions, indicating that Mn and Fe existed in the same intermetallic phase (Al_7_Cu_2_Fe). Therefore, subsequent homogenization is necessary to effectively eliminate element segregation and non-equilibrium phases in as-cast 2219 aluminum alloy to obtain excellent microstructures before the thermo-plastic deformation process.

### 3.3. Dissolution Characteristics of Elements and Phases

**Figure 6:**
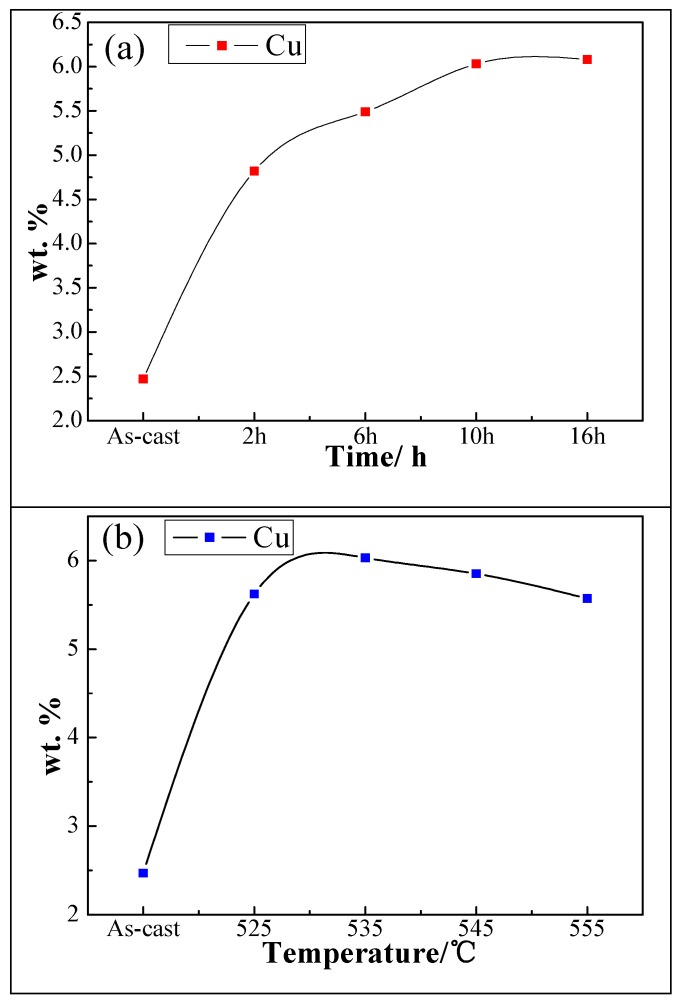
The concentration of Cu in the α (Al) matrix of as-cast and homogenized alloy: (**a**) Cu concentration in α (Al) matrix of homogenized alloy at different second-step time; (**b**) Cu concentration in α (Al) matrix of homogenized alloy at different second-step temperature.

The EDS technique was used to quantitatively analyze and calculate the change of Cu concentration for the various homogenized samples, as shown in Figure 6. The concentration of Cu refers to the average value of the ten different points in the α (Al) matrix, in which the area has the lightest contrast observed in BSEM mode. It can be seen from Figure 6a that the concentration of Cu of as-cast sample was only about 2.47 (wt %), and a continuous increase of Cu concentration was observed with the increase of homogenization time when homogenized at 535 °C. The Cu concentration increased from 2.47 (wt %) of as-cast alloy (without homogenization) up to 6.08 (wt %) of alloy that was homogenized at 535 °C for 16 h, implying the dissolution of the primary eutectic structures during homogenization. It can also be noted from Figure 6b that the Cu concentration increased rapidly when the alloy was treated at 525 °C for 10 h, and the value was 2.28 times larger than that observed in as-cast alloy. The Cu concentration increased slowly when treated at 535 °C, and reached the highest value of 6.08 (wt %). The Cu re-dissolved into the triple conjunctions and grain boundaries due to the occurrence of minor overburn when the homogenization temperature was higher than 545 °C, resulting in a slight reduction of the Cu concentration in the α (Al) matrix. The re-dissolved Cu existed in the form of loose Cu-rich phases in the grain boundaries, which was confirmed in a previous study [25]. Moreover, as seen from the slopes in the Figure 6a,b, the homogenization temperature had a pronounced effect on the change of Cu concentration compared to the influence of time.

**Figure 7:**
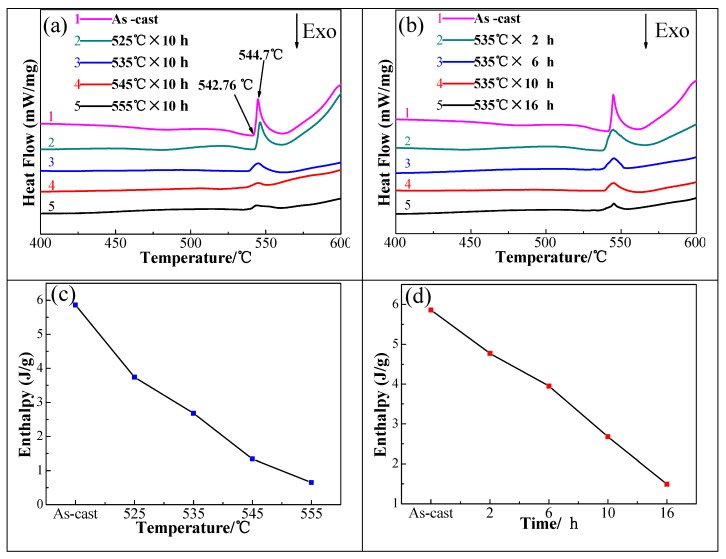
Differential scanning calorimetry (DSC) results of as-cast and homogenized alloy: (**a**) DSC curves of as-cast ingot and specimens homogenized for 10 h at different temperature; (**b**) DSC curves of as-cast ingot and specimens homogenized at 535 °C for different time; (**c**) Temperature and enthalpy values corresponding to (**a**); (**d**) Time and enthalpy values corresponding to (**b**).

To illustrate the phase dissolution of various homogenized regimes, the DSC curves and the corresponding enthalpy values of 2219 aluminum alloy are discussed as shown in Figure 7. Under the same experimental conditions, the phase composition determines the peak of temperature [26]. The area is related to the phase volume fraction under a given endothermic dissolution peak, and the phase volume fraction could be calculated qualitatively by its enthalpy value, as discussed by [3,27,28,29].

Only one relatively high endothermic peak was present in the as-cast alloy (curve 1 in Figure 7a,b), at 544.7 °C. The enthalpy value related to the endothermic peak was identified at about 5.86 J/g in the DSC graph of the as-cast alloy (Figure 7c,d), corresponding to the dissolution of eutectic α (Al) + Al_2_Cu. Further investigations show that the non-equilibrium solidification eutectic should start melting at about 542.7 °C. The endothermic peak at about 544.7 °C disappeared obviously (Figure 7a,b), demonstrating a significant reduction in the amount of non-equilibrium phases α (Al) + Al_2_Cu after homogenization at 535 °C for 10 h, and the enthalpy of the alloy that was homogenized at 535 °C for 10 h was only about 2.68 J/g. This further supports the exceedingly good effect of homogenization treatment in the present paper. Therefore, the upper limit temperature of homogenization was 542.7 °C. The enthalpy value related to the endothermic peak gradually reduced with increasing homogenization temperature or prolonged holding time, indicating the dissolution of some non-equilibrium phases during homogenization [4,28,29].

### 3.4. Microstructure of Homogenized Alloys

**Figure 8:**
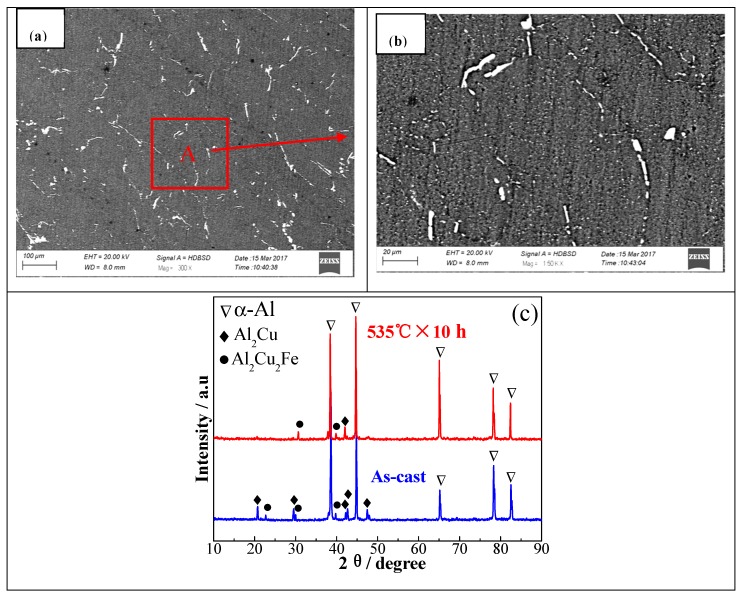
Microstructures and XRD spectra of 2219 aluminum alloy homogenized at 535 °C: (**a**) 535 °C × 10 h; (**b**) the corresponding local magnified map from Figure 5a; (**c**) X-ray diffraction patterns of the as-cast and homogenized alloy.

The microstructures and XRD spectra of 2219 aluminum alloy homogenized at 535 °C are shown in Figure 8. It can be noted from Figure 8a,b that the dendritic-network structures were eliminated increasingly in the volume fraction and the massive residual phases became smaller and sparser. It is also clearly confirmed from Figure 8c that the primary eutectics were mainly comprised of α (Al), Al_2_Cu, and Al_7_Cu_2_Fe phases in the as-cast alloy, which was coincident with EDS results (Figure 4c,d). The diffraction peaks of the non-equilibrium phase Al_2_Cu along the grain boundaries decreased significantly when the specimen was homogenized at 535 °C for 10 h, indicating that the primary eutectic phase inevitably dissolved into the matrix. And the diffraction peaks of the necklace-like phase Al_7_Cu_2_Fe have changed a little during homogenization treatment. The local magnified area A also verified the microstructural behaviors, as shown in Figure 8b. Therefore, it is indicated that the present technological procedure (535 °C × 10 h) was suitable for 2219 aluminum alloy in industrial production.

**Figure 9:**
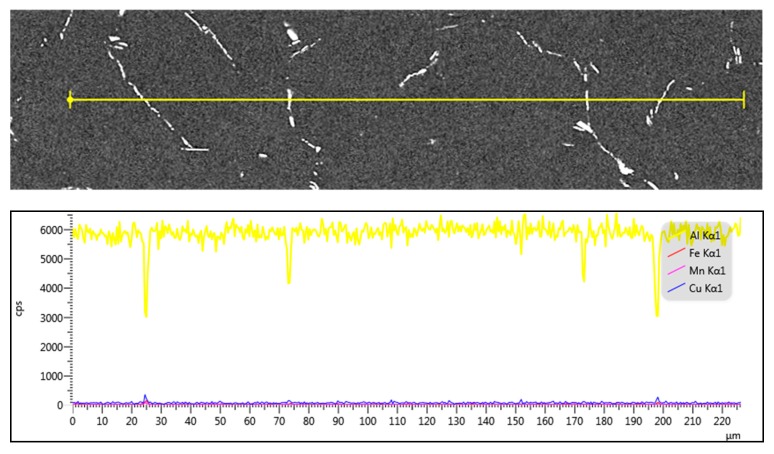
BSEM image and elements line-scanning of alloy homogenized at 535 °C × 10 h.

Figure 9 describes the line scanning results of the homogenized 2219 aluminum alloy. It can be seen that the segregation degrees of the main elements Cu, Mn, and Fe were obviously decreased and the concentration of the elements became homogeneous compared with the as-cast microstructure. However, there was still slight segregation of Al, Cu, and Fe in the necklace-like residual phases or Cu-rich phases, which fits well with the EDS and XRD results (Figure 4 and Figure 8). This is probably owing to the low diffusion velocity [18,23] and the high content of Cu in the 2219 aluminum alloy (about 6.2% Cu), which is slightly higher than the critical solid solubility of elemental Cu in the matrix homogenized at 535 °C [24].

The water quenching of large-size industrial ingots directly after homogenization treatment is likely to cause the emergence of cracking, incomplete hardening, and other defects which have negative effects on the quality of the deformed products. One possible approach was designed when good effect, low energy consumption, and easy operation were taken into account, when the homogenized 2219 alloy ingots were first cooled to the deformation temperature (usually 420–480 °C), then directly multiple-axes forged, ring rolled, and on-line quenched in industrial production. Detailed processes of direct deformation after homogenization treatment for 2219 alloy should be developed in a further study.

### 3.5. Response Behaviors of Microstructure

#### 3.5.1. SEM and TEM Observations

**Figure 10:**
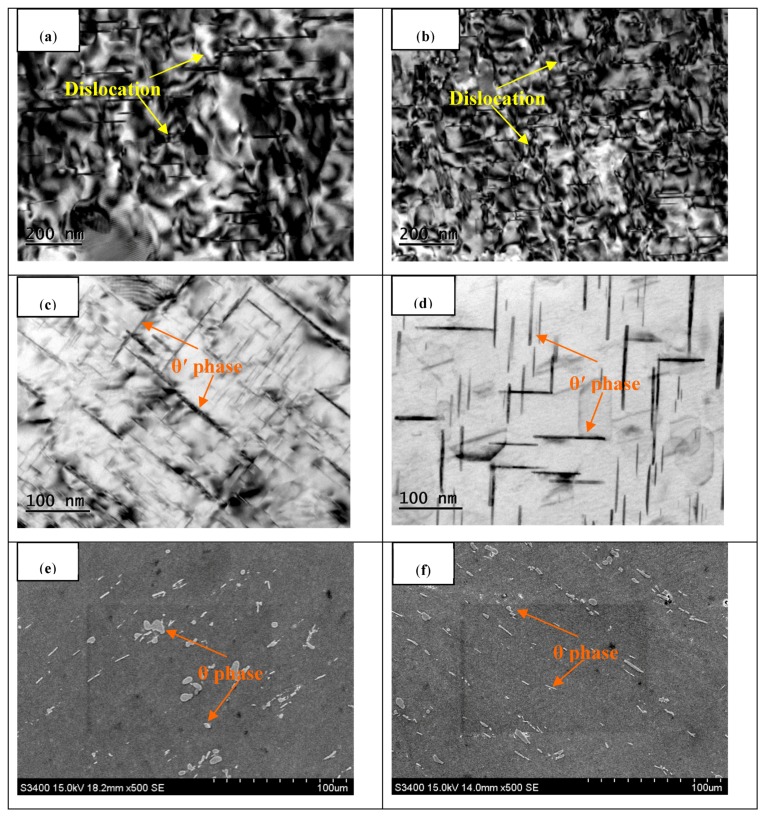
Microstructure evolution of 2219 aluminum alloy for as-cast and homogenized sample with or without T6 treatment after forging, (**a**,**b**) TEM micrographs of as-cast (**a**) and homogenized (**b**) sample after forging; (**c**,**d**) TEM micrographs of as-cast (**c**) and homogenized (**d**) sample with T6 treatment after forging; (**e**,**f**) SEM micrographs of as-cast (**e**) and homogenized (**f**) sample with T6 treatment after forging.

The microstructure evolution of 2219 aluminum alloy forging subjected to hot deformation, with or without T6 treatment for as-cast and homogenized samples are shown in Figure 10. It can be seen that the substructures were developed from dislocation networks during the plastic deformation, which originated from the existence of dislocation climbing, sliding, and tangling. The sub-boundaries were generated by the accumulation of dislocations, having a particular negative influence on the dislocation movements and driving force to deformations. Previous studies have shown that the number density of dislocations has a profound effect on the population of θ′ precipitates [2,4,7,8]. The number density of dislocation networks evidently increased after the homogenization and hot deformation (Figure 10b), as did the number density of θ′ precipitates for the forged and T6-treated sample with homogenization treatment (Figure 10d). Figure 10c shows the coarse and unevenly distributed θ′ precipitates after T6 treatment for the nonhomogenized specimen. In contrast, the precipitated phase θ′ precipitates that formed in the homogenized 2219 aluminum alloy became more heterogeneously distributed in size and highly populated in number after T6 treatment, as observed in Figure 10d. The hindering effect of these dense θ′ precipitates is the main reason for the strengthening of the T6 treated alloy, which restricts the motion of dislocations and the migration of subgrain boundaries within the alloy [2,4,7,8]. It was also noted from Figure 10e that a large number of short rod-like or block impurity phase particles and white fine dispersed θ (Al_2_Cu) phases agglomerated along the maximum strain direction of the as-cast sample. As observed, the volume fraction of the impurity phases became smaller, and the distribution of the white dispersed θ (Al_2_Cu) phases became more uniform after homogenization treatment, as shown in Figure 10f. Accordingly, it can be declared that the homogenization treatment contributed to further degrees of precipitated phase and grain refinement in the multiple axes forging and ring rolling processes.

#### 3.5.2. EBSD Analysis

**Figure 11:**
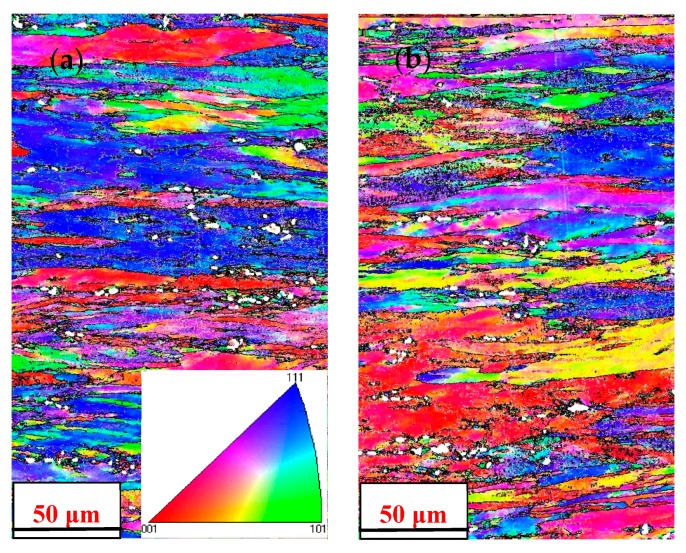

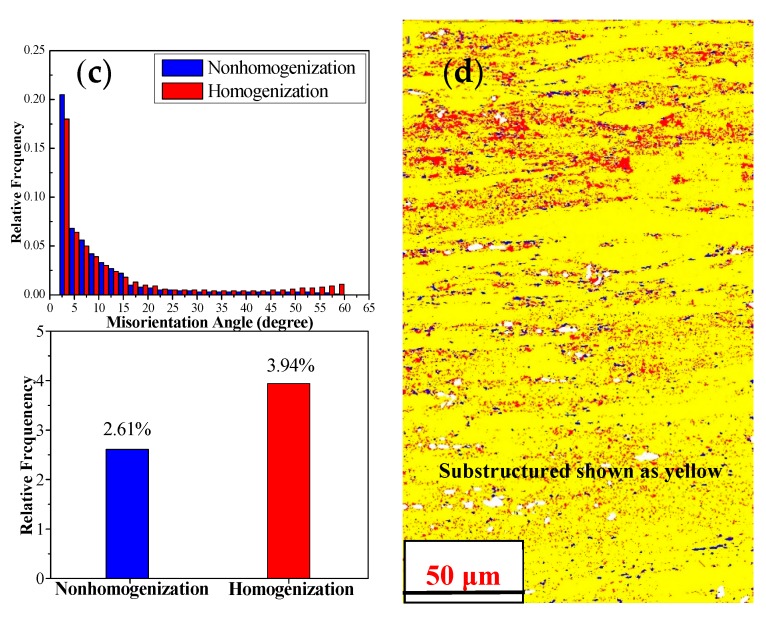
Electron backscattered diffraction (EBSD) images of grain structure for the two different homogenized samples after hot deformation: (**a**) orientation map and grain boundary map of nonhomogenized sample after forging; (**b**) orientation map and grain boundary map of homogenized sample after forging; (**c**) misorientation angle distribution and the recrystallized fraction of nonhomogenized and homogenized sample after forging; (**d**) the corresponding recrystallization map of homogenized sample after forging.

Figure 11 presents the EBSD orientation map, the grain boundaries map, the misorientation angle distribution, and the corresponding recrystallization map of the homogenized and nonhomogenized 2219 alloy subjected to multiple axes forging and ring rolling. Generally, the boundaries with misorientation <2° were described as deformed grain; the misorientation between 2° and 15° were described as sub-grain, which were related to low-angle grain boundaries (shown as a white line); and those with misorientation above 15° were described as recrystallized grain, which were related to high-angle grain boundaries (shown as a black line) [30,31,32]. Figure 11a,b show the orientation imaging microscopy (OIM) images from the hot-deformed specimen before T6 treatment, indicating randomly-oriented elongated grains. It can be observed from Figure 11a,b that the microstructures were almost covered with the fibrous elongated grains. The EBSD analyses showed that a substructure with low misorientation formed during rolling before T6 treatment, which was confirmed by the TEM analyses (Figure 10a,b). A typical recovered grain structure is for the interior of the elongated grains to have a high density of subgrains. However, mainly due to size restrictions and the limitation of scanning resolution, these subgrains could not be observed clearly in the EBSD analyses. There were few isolated recrystallized grains distributed randomly along the elongated grain boundaries. As seen from Figure 11c, the volume fraction of the high-angle grain boundaries (HAGBs) was 54.7% for the nonhomogenized alloy. When the alloy was subjected to homogenization treatment, a more refined grain was seen and the microstructure was still composed of elongated grains, as shown in Figure 11b. The volume fraction of HAGBs was 8.8% higher than that of the alloy without homogenization treatment. Large-angle grain boundaries were transformed from low-angle grain boundaries by incorporating and absorbing dislocations. The fraction of HAGBs increased by employing homogenization, which indicated that more recrystallized grains were formed. Similar observations of the increase of the fraction of HAGBs with the application of homogenization treatment were also reported in Al-Cu alloys [13]. Figure 11b–d provide the grain boundaries map, misorientation angle distribution, and the corresponding recrystallization map of the homogenized sample, respectively. It can be seen that the misorientation angles were mostly less than 15 °, associated to the substructure (Figure 11b,d). The inset at the bottom half of Figure 11c is the volume fraction of dynamic recrystallization for the homogenized sample, the microstructure characteristics mainly exhibited deformed structure (red color) and substructure (yellow color), and only about 3.94% recrystallized grains (blue color in Figure 11d) existed in the homogenized as-deformed sample. The recrystallized fraction of nonhomogenized sample during deformation process was less, about 2.61%.

**Figure 12:**
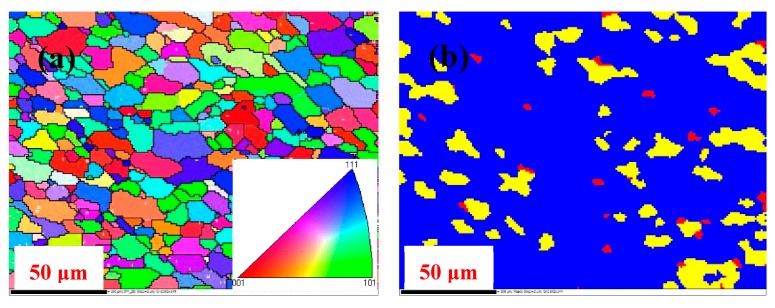

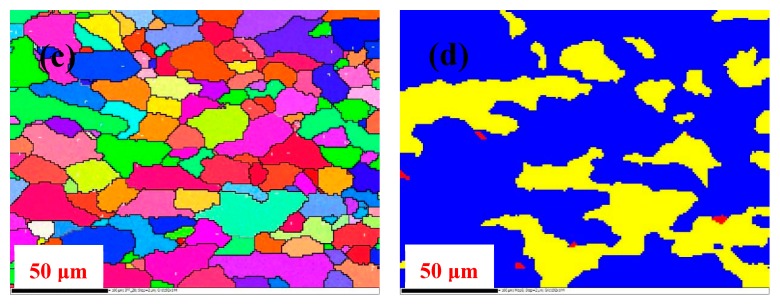
EBSD images of grain structure for the two different homogenized samples subjected to hot deformation and T6 treatment: (**a**) orientation map and grain boundary map of homogenized sample with T6 treatment after forging; (**b**) the corresponding recrystallization map of forged and T6-treated sample (with homogenization); (**c**) orientation map and grain boundary map of nonhomogenized sample with T6 treatment after forging; (**d**) the corresponding recrystallization map of forged and T6-treated sample (without homogenization).

To further clarify the effect of homogenization schemes on the recrystallization behavior, the hot-deformed samples were treated by the T6 treatment. The grain sizes and recrystallization behavior for T6 treated 2219 alloy with and without homogenization treatment are shown in Figure 12. All samples exhibited main quasi-equiaxed grains and a higher fraction of HAGBs, which is a typical recrystallization microstructure. The measured recrystallized volume fraction and grain size for nonhomogenized specimens were 70.7% and 25.7 μm, respectively. It is apparent that the recrystallized volume fraction was higher and the grain size was smaller in the material with homogenization treatment (84.5% and 17.9 μm, respectively). Additionally, a slight increase in HAGBs, represented as black lines in Figure 12a,c, was also observed after homogenization treatment. Thus, the homogenization treatment improved the formation of fine recrystallized grain.

### 3.6. Influence of the Microstructural Response on the Mechanical Properties

#### 3.6.1. Mechanical Properties of Alloy Forging

**Figure 13:**
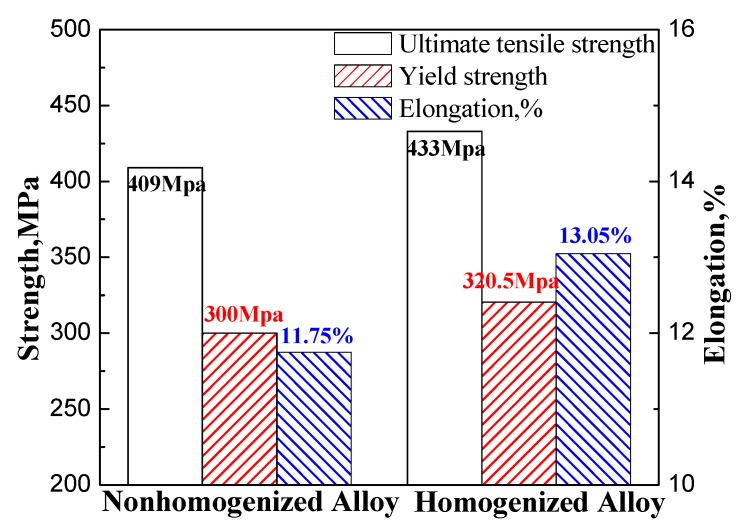
Mechanical properties of 2219 aluminum alloy for as-cast and homogenized sample after forging and T6 treatment.

Figure 13 shows the mechanical properties of 2219 aluminum alloy forgings subjected to hot deformation and T6 treatment for as-cast and homogenized samples. The ultimate tensile strengths of the forgings in the case of no homogenization and homogenization treatment were 409 MPa and 433 MPa, respectively. Evidently, the strength of the homogenized alloy was observed to increase by 5.87% after homogenization at 535 °C for 10 h. A similar increase behavior was also observed in the yield strength: from 300 to 320.5 MPa after the homogenization process. In addition, a higher elongation % was also observed for the homogenized alloy compared to alloys without homogenization after the deformation and T6 treatment processes. Note that the elongation increased by 11.07% after the homogenization treatment.

#### 3.6.2. Strengthening Mechanism of Alloy Forging

The change of strength can be attributed to the microstructural evolution during homogenization. Evidently, the homogenization procedure encouraged numerous brittle eutectic phases to effectively dissolve into the matrix, reducing the probability of brittle fracture along grain boundaries and improving the strength of the material. Meanwhile, solid solution strengthening can be achieved by non-equilibrium solution [21,25], and thereby it was beneficial to obtain aging strengthening through precipitated phases precipitating from the matrix during the subsequent aging process. Besides, the distinct microstructural changes during ring rolling, which were characterized by high density dislocations (Figure 10b) and fine sub-grains with relatively low misorientations (Figure 11d) were responsible for the increase of tensile strength, as reported previously [28,33]. Highly-dense dislocations and fine subgrain boundaries can efficiently enhance homogeneous nucleation and precipitation of the θ′ phases, and these precipitates were found to exist together with the dislocations and therefore improved the strength of the alloy. Furthermore, the improvement of the tensile strength can also be attributed to finer grain size, the fragmentation of θ phase (Al_2_Cu), higher density of uniformly scattered θ′ phase (Al_2_Cu), and higher fraction of the HAGBs, as discussed in the microstructural evolution section (Figure 10 and Figure 11). The enhancing behavior of the strength was in good accordance with that researched by Ibrahim et al. [12,13], who revealed significant improvements in the mechanical properties and deformation homogeneity due to microstructural refinement during homogenization. Meanwhile, the increase of the elongation can be explained by the microstructural evolution in Figure 8 and Figure 10, Figure 11 and Figure 12, in which the brittle dendrites and solute segregation were reduced in number and the microstructure became more homogeneous for the alloy subjected to homogenization. Another reason is the microstructural transformation from the dendritic to quasi-equiaxed grain microstructures and the higher average boundary misorientation angle for the homogenized alloys than those of nonhomogenized samples [13,34].

## 4. Conclusions

- Severe dendritic segregation was present in large-size DC-cast Al-Cu-Mn alloy ingots. The main elements Cu, Mn, Fe were largely segregated in the grain boundary. There were massive non-equilibrium eutectic phases in the as-cast alloy, and the dissolvable secondary phase was θ (Al_2_Cu).
- The dendritic-network structures were eliminated sufficiently, the non-equilibrium phases along the grain boundaries became sparser and the distribution of all the elements became more homogenized by using the optimal homogenization process. Besides, the morphology of insoluble compounds Al_7_Cu_2_Fe was not regular and it remained nearly unchanged during homogenization.
- Homogenization can generate more homogenous microstructures with higher number density of dislocation networks, higher density of evenly-distributed θ′ or θ phase (Al_2_Cu), finer grains, higher volume fraction of high-angle grain boundaries, and higher volume fraction of recrystallized grains compared with unhomogenized alloys during rolling and subsequent T6 treatment.
- The tensile strength, yield strength and the elongation of the alloys with homogenization treatment were increased by 5.87%, 6.83%, and 11.07% compared to nonhomogenized alloy. The suitable homogenization processing was determined to be 535 °C × 10 h.
